# A real-world cost-effectiveness analysis of nebulized budesonide and intravenous methylprednisolone in acute exacerbation of chronic obstructive pulmonary disease

**DOI:** 10.3389/fphar.2022.892526

**Published:** 2022-09-13

**Authors:** Yong-Li Gu, Zeng-Xian Sun, Ying Sun, Yan Wen, Xin Guan, Dao-Li Jiang, Cong Cheng, Huan Gu

**Affiliations:** ^1^ Department of Pharmacy, The First People’s Hospital of Lianyungang, Lianyungang, China; ^2^ Department of Respiratory, The First People’s Hospital of Lianyungang, Lianyungang, China; ^3^ School of International Pharmaceutical Business, China Pharmaceutical University, Nanjing, China; ^4^ Department of Pharmacy, The Affiliated Hospital of Xuzhou Medical University, Xuzhou, China; ^5^ Department of Pharmacy, Lianyungang Affiliated Hospital of Nanjing University of Chinese Medicine, Lianyungang, China

**Keywords:** budesonide, methylprednisolone, chronic obstructive pulmonary disease, acute exacerbation, minimum cost analysis, cost-effectiveness analysis

## Abstract

**Objective:** To assess the cost-effectiveness of nebulized budesonide and intravenous methylprednisolone in the treatment of acute exacerbation of chronic obstructive pulmonary disease (AECOPD) in a real-world setting.

**Materials and methods:** Data from 291 patients with AECOPD were collected from the information system of a tertiary hospital in China. Patients were categorized into two groups: those treated with nebulized budesonide (*n* = 148) and those treated with intravenous methylprednisolone (*n* = 143). Clinical efficacy and the rate of no readmission within 1 year after discharge were used as effect indicators, and a cost-effectiveness analysis was conducted from the perspective of the Chinese healthcare system. Logistic regression, generalized linear regression, and bootstrap methods were used for sensitivity analyses.

**Results:** There was no statistical difference between the budesonide and methylprednisolone groups in clinical efficacy rates (94.6% vs. 93.7%). The cost-minimization analysis shows that budesonide is not cost-effective owing to higher total cost. In terms of readmission rates, budesonide was again not cost-effective, with an incremental cost-effectiveness ratio (ICER) of 22276.62 CNY, which is higher than the willingness to pay (WTP) of 20206.20 CNY, the mean per admission expenditure in China. The sensitivity analyses confirm that these results are robust.

**Conclusion:** Compared with intravenous methylprednisolone, nebulized budesonide is not a cost-effective strategy for AECOPD patients in China.

## 1 Introduction

Chronic obstructive pulmonary disease (COPD) is a leading cause of mortality worldwide and entails a significant economic and social burden (Lozano et al., 2012; Vos et al., 2012). In China, COPD is the fifth most common cause of death, accounting for 31.1% of the world’s total deaths from COPD (Yin et al., 2016; GBD 2017 Causes of Death Collaborators, 2018). Acute exacerbation of chronic obstructive pulmonary disease (AECOPD) is an episode of worsening symptoms that negatively impacts health status (Vogelmeier et al., 2017). AECOPD is a frequent cause of hospital admission and accounts for 50%–75% of the health care costs incurred by patients with COPD (Celli et al., 2004).

COPD patients usually experience about 0.5–3.5 exacerbations per year (Hurst et al., 2010). However, greater frequency of exacerbations is associated with accelerated lung function decline (Donaldson et al., 2002), quality of life impairment (Seemungal et al., 1998) and increased mortality (Soler-Cataluña et al., 2005). Systemic corticosteroids (SCs) in COPD exacerbations shorten recovery time and improve lung function (Global, 2021), and a dose of 40 mg prednisone per day for 5 days is recommended for management of exacerbations of COPD (Leuppi et al., 2013). However, because of adverse effects such as osteoporosis and bone fractures, glucose intolerance, and myopathy (Kwong et al., 1987; Decramer et al., 1994; Henzen et al., 2000), nebulized budesonide alone may be a suitable alternative to SCs and provides similar benefits to intravenous methylprednisolone (Ding et al., 2016). Because the cost of budesonide is higher than that of methylprednisolone, the Global Initiative for Chronic Obstructive Lung Disease guideline (GOLD) states that the choice between these options may depend on local cost issues (Global, 2021).

The present real-world study assesses the efficacy, safety, and cost-effectiveness of nebulized budesonide versus intravenous methylprednisolone for AECOPD patients from the perspective of the Chinese healthcare system.

## 2 Materials and methods

### 2.1 Data and sources

For this single-center retrospective study of patients with AECOPD, data collection was carried out from 1 January 2020 to 31 December 2020 at Lianyungang First People’s Hospital, a tertiary general hospital. Data were collected from patients’ electronic medical records and retrieved for analysis in a real-world setting. Eligible patients were aged between 45 and 80 years old, with a confirmed diagnosis of COPD and currently in acute exacerbation. Patients were ineligible if they received SCs in the past month, or had a history of pneumothorax, pulmonary embolism, or other respiratory diseases. Patients were categorized into two groups: those treated with budesonide suspension (2 mg, tid, nebulized inhalation) and those treated with methylprednisolone (40 mg, qd, intravenous infusion). The duration was 5–7 days in both groups.

### 2.2 Effectiveness, safety, and cost assessment

In this study, clinical effectiveness was evaluated by professional clinicians according to the patient’s respiratory symptoms (cough, sputum, and dyspnea), pulmonary function, and blood gas analysis. In addition, to assess the long-term effect, the two groups’ rates of no readmission within 1 year after discharge were compared. Adverse events that occurred during the treatment were also extracted from medical records and investigated.

As the study context is the Chinese healthcare system, only data relating to direct medical costs were considered. The costs of medication, examinations, laboratory tests, ward beds, nursing, and other costs were collected from the hospital information system, and the total direct medical costs for each patient were calculated as the sum of all cost categories. Mean cost per patient over the entire period was calculated by summing the totals and then dividing the sum by the sample size in each arm. Discounting was not considered in this study because of the short time horizon. All resource costs were represented in Chinese yuan (CNY).

### 2.3 Statistical analysis

Continuous variables were presented as mean and standard deviation in each group and compared using a two-tailed Student’s *t*-test if the variables conformed to a normal distribution. In other cases, the minimum, maximum, median, and interquartile ranges were calculated for each group and compared using the Wilcoxon signed-rank test. Categorical variables were presented as count (n) and percentage (%) and compared using Fisher’s exact test or the chi-square test. If a difference in patient characteristics between the two groups was statistically significant, propensity score matching (PSM) was to be used to balance the bias. All analyses were processed using SPSS 23.0 software (IBM), and the significance level was defined as two-sided *α* = 0.05.

Sensitivity analyses were performed to examine the stability and robustness of the results. First, a logistic regression model was used to control confounding factors, and generalized linear regression was used to identify the influence factors on direct medical costs. Second, the bootstrap method was used for repeated sampling (1,000 times) with replacement, and a cost-effectiveness acceptable curve (CEAC) was drawn according to the sampling results.

## 3 Results

### 3.1 Patient characteristics

Patient characteristics are summarized in Table 1. A total of 291 patients were identified, 148 in the budesonide group and 143 in the methylprednisolone group. As all characteristics were balanced between the two groups, PSM was not used.

**TABLE 1:** Baseline characteristics.

Variables	Nebulized budesonide (*n* = 148)	Intravenous methylprednisolone (*n* = 143)	*p*-value
Male, n (%)	117 (79.1)	104 (72.7)	0.207
Mean age, years (SD)	71.61 (9.69)	71.87 (9.01)	0.809
BMI, kg/cm^2^ (SD)	23.50 (6.71)	23.12 (4.03)	0.564
Mean COPD duration, years (SD)	16.93 (14.06)	15.08 (10.76)	0.211
Current smoker, n (%)	92 (62.2)	80 (55.9)	0.281
Mean number of pack-years smoked (SD)	53.8 (26.12)	49.78 (31.23)	0.501
COPD exacerbations in the past 12 months, n (%)	35 (23.6)	37 (25.9)	0.660
oxygen therapy, n (%)	142 (95.9%)	140 (97.9%)	0.231
respiratory support, n (%)	31 (20.9%)	42 (29.4%)	0.097
Comorbidity, n (%)			
Hypertension	39 (26.4)	44 (30.8)	0.404
Diabetes	15 (10.1)	16 (11.2)	0.771
Cerebral infarction	9 (6.1)	16 (11.2)	0.120
peripheral blood eosinophils, cells/μl (SD)	95.0 (102.2)	116.32 (147.9)	
Combination medication, n (%)			
short-acting bronchodilator	127 (85.8)	112 (78.3)	0.095
Theophylline	110 (74.3)	111 (77.6)	0.511
Antimicrobials	144 (97.3)	141 (98.6)	0.434
Arterial blood-gas analysis before treatment (mean ± SD)			
PH	7.39 ± 0.048	7.39 ± 0.032	0.334
PaCO2	54.19 ± 14.90	51.00 ± 16.46	0.280
PaO2	75.71 ± 15.22	74.25 ± 16.38	0.624
Pulmonary functions before treatment (mean ± SD)			
FEV_1_	0.84 ± 0.168	0.82 ± 0.183	0.561
FEV_1_/FVC	52.49 ± 9.60	51.99 ± 9.859	0.832

### 3.2 Effectiveness

#### 3.2.1 Blood gas analysis

Blood gas analysis was carried out for 114 patients, 58 in the budesonide group and 56 in the methylprednisolone group. The results show that PaCO_2_ and PaO_2_ levels improved significantly in both groups. However, after treatment, no significant differences in pH, PaO_2_, or PaCO_2_ levels were found between the groups (Table 2).

**TABLE 2:** Comparison of effectiveness of nebulized budesonide and intravenous methylprednisolone.

Observation indicators	Nebulized budesonide (*n* = 148)	Intravenous methylprednisolone (*n* = 143)	*p*-value
Arterial blood-gas analysis before treatment (mean ± SD)			
PH	7.41 ± 0.045	7.40 ± 0.036	0.185
PaCO2	44.93 ± 9.11*	46.07 ± 7.92*	0.478
PaO2	83.90 ± 13.21*	79.35 ± 13.19*	0.069
Pulmonary functions before treatment (mean ± SD)			
FEV1	1.06 ± 0.268*	1.05 ± 0.240*	0.929
FEV1/FVC	57.59 ± 9.58*	59.71 ± 11.77	0.407
Clinical efficacy rate, n (%)	140 (94.6)	134 (93.7)	0.747
The rate of no readmission within 1 year after discharge, n (%)	110 (74.3)	59 86 (60.1)	0.010
Hospital stay (days)	14.79 ± 7.58	13.83 ± 6.41	0.246

*After treatment versus before treatment, *p* < 0.05

#### 3.2.2 Pulmonary function

Pulmonary function tests were carried out for 70 patients. The results show that the FEV_1_ and FEV_1_/FVC levels improved significantly in both groups. However, after treatment, there were no significant differences in the FEV_1_ and FEV_1_/FVC levels between the groups (Table 2).

#### 3.2.3 Clinical efficacy

The clinical efficacy rates of the budesonide and methylprednisolone groups were 94.6% and 93.7%, respectively, and there was no statistical difference between the groups (*p* = 0.747) (Table 2).

#### 3.2.4 Rate of no readmission

The rate of no readmission within 1 year after discharge in the budesonide group was significantly higher than in the methylprednisolone group (74.3% vs. 60.1%, χ^2^ = 6.655, *p* = 0.010) (Table 2).

### 3.3 Safety

During hospitalization, adverse events that occurred in the two groups were hyperglycemia, oropharynx fungal infection, sleep disorder, and stomach discomfort. Although the rate of adverse events was significantly higher in the methylprednisolone group than in the budesonide group, all the events were relatively mild, and no special treatment was given (Table 3).

**TABLE 3:** Adverse events between the two groups.

Observation indicators, *n* (%)	Nebulized budesonide (*n* = 148)	Intravenous methylprednisolone (*n* = 143)	*p*-value
Hyperglycemia	0 (0)	3 (2.09)	
Oropharynx fungal infection	5 (3.37)	0 (0)	
Sleep disorder	0 (0)	4 (2.80)	
Stomach discomfort	0 (0)	7 (4.89)	
Total	5 (3.37)	14 (9.79)	0.027

After discharge, there were no adverse events occurred in the two groups.

### 3.4 Pharmacoeconomic evaluation

#### 3.4.1 Cost-minimization analysis

As there was no statistical difference between the groups in clinical efficacy rates, cost-minimization analysis (CMA) was used. Total costs and a breakdown of the categories are given in Table 4. For most of the costs, including medication, examinations, laboratory tests, treatment, diagnosis, nursing, rehabilitation, and medical consumables, there were no significant differences between the groups. The total costs were significant higher for the budesonide group than for the methylprednisolone group (20460.56 CNY vs. 17297.28 CNY, *p* = 0.037). Therefore, the CMA results suggest that intravenous methylprednisolone is more economical than nebulized budesonide in the treatment of AECOPD.

**TABLE 4:** Comparison of hospitalization costs (CNY) for nebulized budesonide and intravenous methylprednisolone groups.

Cost, mean (SD)	Nebulized budesonide (*n* = 148)	Intravenous methylprednisolone (*n* = 143)	*p*-value
Total hospitalization cost	20460.56 (13870.53)	17297.28 (11716.82)	0.037
Total medication cost	8074.09 (9849.04)	6210.41 (6223.48)	0.056
Western medcine	7998.20 (9816.17)	6110.69 (6126.78)	0.051
Chinese patent drug	39.95 (89.16)	40.34 (156.38)	0.979
Chinese herbal medicine	36.21 (175.70)	59.36 (290.22)	0.409
Imaging examination cost	583.68 (554.55)	579.59 (553.07)	0.950
Laboratory test cost	2650.98 (1081.39)	2409.72 (1088.52)	0.059
Treatment cost	2260.77 (2712.92)	1890.60 (2540.18)	0.231
Diagnostic cost	1589.94 (1808.00)	1356.73 (1763.78)	0.267
Ward bed cost	1037.35 (1040.61)	841.50 (931.18)	0.092
Nursing cost	476.09 (283.97)	449.63 (349.39)	0.478
Rehabilitation cost	11.93 (42.94)	23.44 (97.17)	0.190
Medical consumables cost	676.54 (962.54)	539.75 (617.99)	0.152
Other cost	1193.96 (1534.34)	1601.70 (1685.57)	0.032

#### 3.4.2 Cost-effectiveness analysis

The rate of no readmission within 1 year after discharge was then adopted as the effect indicator, and a cost-effectiveness analysis (CEA) was used. The results are shown in Table 5. The ICER value of the budesonide group was 22276.62 CNY; thus, compared with intravenous methylprednisolone, nebulized budesonide cost 22276.62 CNY extra and saved one readmission within 1 year after discharge.

**TABLE 5:** Base case results of the cost-effectiveness analysis.

Group	Cost (C)	Effectiveness (E)	I (%) CER (ΔC/ΔE)
Nebulized budesonide	20460.56	74.3	22276.62
Intravenous methylprednisolone	17297.28	60.1	

## 4 Sensitivity analysis

For the logistic regression analysis, clinical efficacy and readmission within 1 year after discharge were used as dependent variables, and all possible influencing factors were included as independent variables. Consistent with the results of the basic analysis, the choice of budesonide or methylprednisolone as the main drug had no significant effect on clinical efficacy but had a significant effect on readmission within 1 year after discharge (Table 6).

**TABLE 6:** Logistic regression analysis of treatment effect.

Factors	Clinical efficacy		Readmission within 1 year after discharge	
	Coefficients	*p*-value	Coefficients	*p*-value
Sex	0.207	0.711	−0.19	0.609
Age	−0.005	0.863	−0.026	0.150
Comorbidity	−1.082	0.198	−0.175	0.570
short-acting bronchodilator	0.612	0.550	−0.498	0.317
Theophylline	−1.146	0.102	0.260	0.496
Antimicrobials	−24.159	0.998	0.948	0.510
COPD duration	−0.066	0.025	−0.015	0.296
Treatment option	0.092	0.910	−0.753	0.019
Constant	6.124	0.078	1.83	0.157

Because the costs did not follow a normal distribution, generalized linear model analyses were used to examine the associated factors for direct medical and medication costs. Total hospitalization and total medication costs were taken as dependent variables respectively, and all possible influencing factors were included as independent variables. The gamma distribution was used to perform generalized linear regression with identity as the connection function. The results indicate that patients treated with budesonide had greater total hospitalization and total medication costs (Table 7).

**TABLE 7:** Multiple linear regression model for hospitalization costs.

Factors	Total hospitalization cost		Total medication cost	
	Coefficients	*p*-value	Coefficients	*p*-value
Sex (female = 0)	607.985	0.422	450.870	0.320
Age	−25.837	0.482	−8.567	0.652
Comorbidity (no = 0)	1151.157	0.073	1530.475	0.001
Antimicrobials received (no = 0)	1824.257	0.536	1453.217	0.421
COPD duration	15.178	0.278	−6.750	0.520
Hospital days	1160.306	<0.0001	602.475	<0.0001
Treatment option (budesonide = 0)	−1336.095	0.036	−1107.915	0.006
Constant	3112.654	0.256	−1098.050	0.680

**FIGURE 1:**
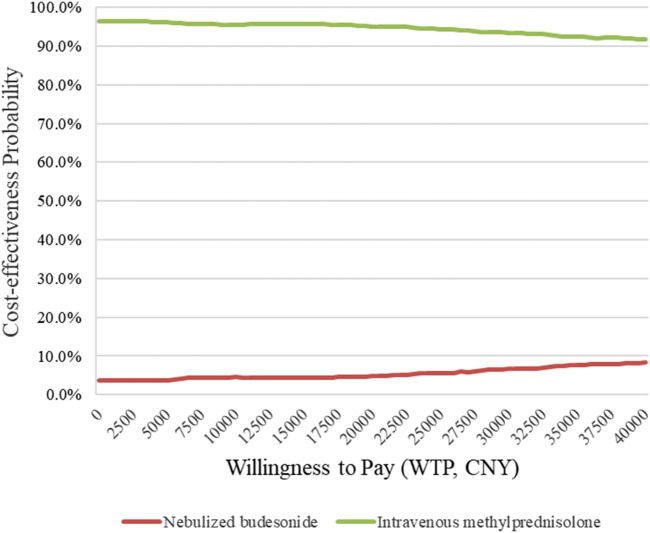
CEAC by bootstrap analysis on the clinical efficacy indicator.

**FIGURE 2:**
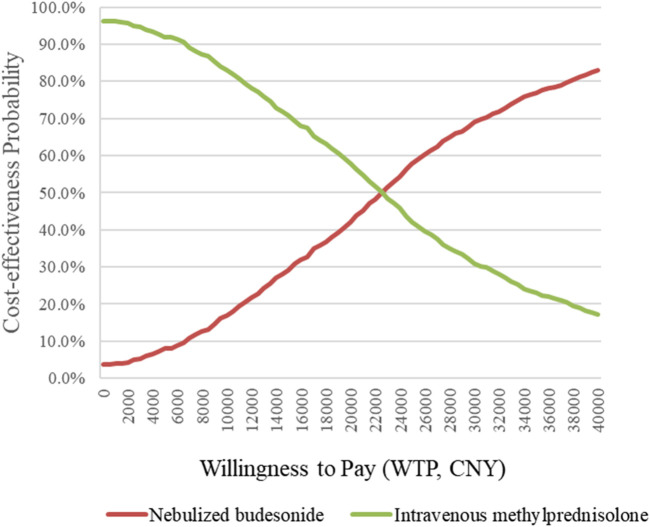
CEAC by bootstrap analysis on the rate of no readmission indicator.

Because this study is retrospective, bootstrapping was used to reduce sampling error, and a CEAC was drawn. When the clinical efficacy rate was used as the effect indicator, the results show that the probability of methylprednisolone being cost-effective at a willingness to pay (WTP) of 40,000 CNY was more than 90% (Figure 1). However, when the rate of no readmission within 1 year was used as the effect indicator, and the WTP was more than 23,000 CNY, nebulized budesonide became cost-effective (Figure 2). These findings are consistent with the results of the basic analysis, which indicates that the analysis is robust.

## 5 Discussion

To our knowledge, this is the first study to use real-world patient-level data to investigate the differences in clinical effectiveness and pharmacoeconomics between nebulized budesonide and intravenous methylprednisolone in the treatment of AECOPD. There are three main findings.

First, for hospitalized patients with AECOPD, nebulized budesonide and intravenous methylprednisolone both improved lung function and blood gas analysis and had similar clinical efficacy. These results are in agreement with the findings of Ding et al. (2016) and the recommendations of expert consensus in China (Cai et al., 2014).

Second, in addition to clinical efficacy, this study also explored the effect of the two interventions in reducing the risk of readmission, which is an important indicator for evaluating the long-term effect on COPD patients. Readmission rates within 1 year after discharge were relatively high for both groups, at 25.7% in the budesonide group and 39.9% in the methylprednisolone group. A history of exacerbations is the most reliable predictor of exacerbations in COPD patients (Donaldson and Wedzicha, 2006; Hurst et al., 2010) and the patients in this study were therefore likely to be readmitted for acute exacerbations. This result may therefore be related to the frequent-exacerbation phenotype of hospitalized patients, especially in China, where most patients are already in group D according to the GOLD guidelines when they first visit a doctor.

Third, in economic terms, when clinical efficacy was used as the short-term efficacy indicator, the CMA results indicated that intravenous methylprednisolone was cost-effective due to lower total costs. However, from a long-term perspective, the CEA results indicated that nebulized budesonide cost 22,276.62 CNY extra compared with intravenous methylprednisolone, while saving one readmission within 1 year after discharge. Therefore, an average cost of one hospitalization for an acute exacerbation can be used as the threshold for WTP. A number of studies have found that AECOPD contributes significantly to the costs of COPD (Rutten-van Mölken et al., 1999; Hilleman et al., 2000; Miravitlles et al., 2002; Toy et al., 2010). In China, a large-scale retrospective study conducted by Liang et al. found that mean expenditure per admission increased from 19,760 CNY in 2009 to 20,118 CNY in 2017 (a growth rate of 0.11%) (Liang et al., 2020). On this trend, by 2021 mean expenditure per admission would have reached 20,206.20 CNY. In terms of willingness to pay, nebulized budesonide was not cost-effective compared with intravenous methylprednisolone because the ICER (22,276.62 CNY) was higher than the WTP (20,206.20 CNY).

A key strength of the present study is that it uses real-world data that are likely to provide reasonably good estimates of absolute event probabilities and costs in actual clinical practice. In addition, for a more comprehensive assessment, its economic evaluation includes different effect indicators, namely, clinical efficacy and no readmission within 1 year after discharge, which correspond to short-term and long-term effects, respectively.

However, the limitations of this study should be noted. First, because it is a single-center retrospective study, the sample size is small and the data identified from the electronic medical record database were incomplete. Second, the analysis focused on direct medical costs and paid no attention to direct nonmedical or indirect costs, which may have influenced the results. Third, the use of mean expenditure per admission, without considering the negative impact of readmission on patients’ lung function and quality of life, may have led to an underestimation of the WTP threshold.

In conclusion, compared with intravenous methylprednisolone, nebulized budesonide is not a cost-effective strategy in terms of either short-term or long-term effect. Large-scale multicenter studies are required to validate the findings of this study.

## Data Availability

The original contributions presented in the study are included in the article/Supplementary Material, further inquiries can be directed to the corresponding author.
